# Inflammatory bowel disease is causally related to irritable bowel syndrome: a bidirectional two-sample Mendelian randomization study

**DOI:** 10.3389/fmed.2023.1166683

**Published:** 2023-04-17

**Authors:** Haoran Ke, Zitong Li, Qianyun Lin, Zefeng Shen, Ye Chen, Jinjun Chen

**Affiliations:** ^1^Hepatology Unit, Department of Infectious Diseases, Nanfang Hospital, Southern Medical University, Guangzhou, China; ^2^Guangdong Provincial Key Laboratory of Gastroenterology, Department of Gastroenterology, Nanfang Hospital, Southern Medical University, Guangzhou, China; ^3^Department of Gastroenterology, Beijing Friendship Hospital, Capital Medical University, Beijing, China; ^4^Department of Urology, Sun Yat-sen Memorial Hospital, Sun Yat-sen University, Guangzhou, Guangzhou, China; ^5^Department of Gastroenterology, Integrative Microecology Center, Shenzhen Hospital, Southern Medical University, Shenzhen, China

**Keywords:** irritable bowel syndrome, inflammatory bowel disease, Mendelian randomization, causal relationship, genome-wide association

## Abstract

**Introduction:**

Inflammatory bowel disease (IBD) and irritable bowel syndrome (IBS) are lifelong digestive diseases that severely impact patients’ quality of life. The existence of a causal association between IBS and IBD remains unclear. This study aimed to determine the direction of causality between IBD and IBS by quantifying their genome-wide genetic associations and performing bidirectional two-sample Mendelian randomization (MR) analyses.

**Methods:**

Genome-wide association studies (GWAS) among a predominantly European patient cohort identified independent genetic variants associated with IBS and IBD. Two separate databases (a large GWAS meta-analysis and the FinnGen cohort) for both IBS and IBD were consulted to retrieve statistics on instrument-outcome associations. MR analyses included inverse-variance-weighted, weighted-median, MR-Egger regression, MR Pleiotropy RESidual Sum and Outlier (MR-PRESSO) methods, and sensitivity analyses were performed. The MR analyses were carried out for each outcome data, followed by a fixed-effect meta-analysis.

**Results:**

Genetically predicted IBD was associated with an increased risk of IBS. Odds ratios (95% confidence intervals) for samples of 211,551 (17,302 individuals with IBD), 192,789 (7,476 Crohn’s disease cases), and 201,143 (10,293 ulcerative colitis cases) individuals were 1.20 (1.00, 1.04), 1.02 (1.01, 1.03), and 1.01 (0.99, 1.03), respectively. After outlier correction using MR-PRESSO, the odds ratio for ulcerative colitis was 1.03 (1.02, 1.05) (*p* = 0.001). However, an association between genetically influenced IBS and IBD was not identified.

**Discussion:**

This study confirms that IBD is causally related to IBS, which may interfere with the diagnosis and treatment of both diseases.

## 1. Introduction

Irritable bowel syndrome (IBS) and inflammatory bowel disease (IBD), including ulcerative colitis (UC), and Crohn’s disease (CD), are chronic and recurrent gastrointestinal disorders. They are highly prevalent and severely impact patients’ quality of life (1–3). The high prevalence rates of IBS and IBD are a significant financial burden on healthcare systems and society, with more than 4.1% of persons worldwide having IBS (3) and more than 0.3% of persons worldwide having IBD (4). IBD and IBS have similar symptoms and demographic characteristics (5, 6). For example, the abdominal pain and diarrhea are predominant symptoms for patients with IBS or IBD, patients with IBD have more severe gastrointestinal symptoms compared with IBS generally; meanwhile inflammation of the gut is a key characteristic of IBD but not IBS. Their etiologies are not fully understood, but both involve genetic, neurological (gut-brain axis), psychological (e.g., stress and anxiety), and environmental factors (e.g., diet) (7–11).

According to a recent meta-analysis, the incidence of IBS-type symptoms (IBS-TSs) was higher in patients with IBD (12). Other studies have suggested that, rather than coexisting with IBS, these symptoms are manifestations of latent inflammation (13). However, symptoms are not always correlated with inflammation. Up to 25.8% of patients with IBD in deep endoscopic/histological remission have reported IBS-TSs (12, 14, 15); this number was significantly higher than that of IBS itself (16). It is important to note that IBS might coexist in patients with an established diagnosis of IBD due to its high prevalence and the inflammation of IBD can cause damage to the nerves and muscles in the intestinal wall, which also lead to IBS-type symptoms. Thus, symptoms alone may not provide enough information to differentiate IBS-type symptoms from ongoing IBD activity, which hampers clinical decisions making regarding treatment (17, 18). In addition, due to the chronic nature of these diseases, it is difficult to determine cause and effect. It is common for patients with IBD to be first diagnosed with IBS based on their clinical symptoms (19), psychological disorders also can affect patients with clinically well-controlled IBD (20). Moreover, residual confounding and reverse causality are inherent problems with cross-sectional and case–control research; thus, no clear causal connection has been identified so far between IBS and IBD.

**Figure 1:**
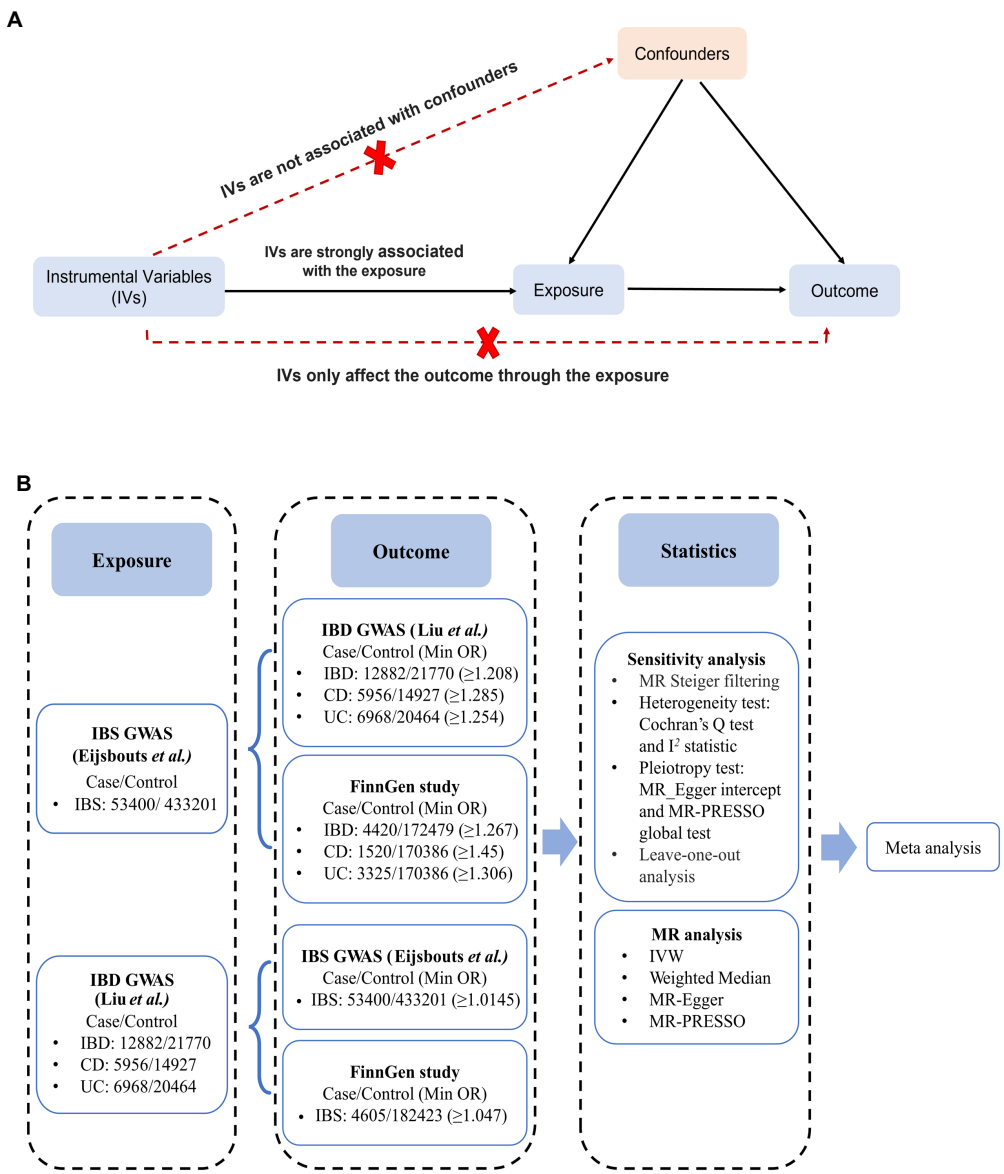
Schematic overview of the study. **(A)** An illustration of Mendelian randomization (MR). The MR design is based on three assumptions: (1) the genetic instrumental variables (IVs) are strongly associated with exposure, (2) IVs only affect outcomes through exposure, and (3) IVs are not associated with any measured or unmeasured confounders. **(B)** MR study from IBS to IBD: independent IBS/IBD-correlated SNPs were used as IVs, whereas SNPs-IBD/IBS data were obtained separately from the IBD GWAS (Liu et al.) /IBS GWAS (Eijsbouts et al.) and the FinnGen cohort. MR and sensitivity analyses were performed for each outcome IBD/IBS data, and the MR analyses from the same exposure were then meta-analyzed to obtain pooled estimates. CD, Crohn’s disease; CI, confidence interval; IBD, inflammatory bowel disease; IBS, irritable bowel disease; IVW, inverse variance weighted; MR-PRESSO, MR Pleiotropy RESidual Sum and Outlier; UC, ulcerative colitis.

Mendelian randomization (MR), an effective method of causal inference (21), stimulates a randomized controlled trial by using genetic variation as an instrumental variable (IV) to assess whether lifetime exposure is causally related to outcomes. Its advantages include minimizing confounding factors and reducing reverse causality. Genetic variation is assigned randomly at conception and therefore does not depend on the environment, and it cannot be altered by disease development or progression (Figure 1A) (21).

This study aims to determine the putative direction of causality between IBS and IBD by quantifying their genome-wide genetic associations and performing bidirectional MR analyses.

## 2. Materials and methods

An assessment of IBD and IBS causality was conducted using bidirectional two-sample MR. Figure 1 shows the specific data sources and research design. The genome-wide association study (GWAS) aggregate data statistics are publicly available, and each GWAS was approved by the appropriate ethics committee. Therefore, additional informed consent and ethical approval were not needed. This study is reported in accordance with the STROBE MR guidelines (22). From the largest GWAS of European ancestry, the major single-nucleotide polymorphisms (SNPs) correlated with IBS or IBD were derived as IVs. Outcome-associated data were extracted from large-scale GWAS meta-analysis data and FinnGen cohort. To avoid overlapping the sample data sources, the UK Biobank data was not retrieved separately.

### 2.1. Sources of genetic IVs

The genetic datasets used in this study were summarized in Supplementary Table S1. The GWAS statistics of IBS were extracted from the meta-analysis performed by Eijsbouts et al. (23),^1^ that utilized data from the Bellygenes Initiative (including the HUNT Study, TWINGENE, Lifelines, Mayo Genome Consortia, Genetic Epidemiology Research on Aging, Estonian Genome Center at the University of Tartu, and Michigan Genomics Initiative) and UK Biobank (cases/controls for IBS: 53,400/433,201) (Figure 1B). The diagnostic criteria for The HUNT Study, TWINGENE, and Lifelines were based on Rome III criteria. The diagnostic criteria for Mayo Genome Consortia and Genetic Epidemiology Research on Aging was an ICD9 code of IBS (564.1) while the Estonian Genome Center at the University of Tartu and Michigan Genomics Initiative used an ICD10 code for IBS (K58) as diagnostic criteria. The details of demographics of Bellygenes cohorts are shown in Supplementary Table S2. The UK Biobank determined IBS according to the inclusion of one or more of the following standards: symptom criteria for a Rome III IBS diagnosis with no alternative diagnostic explanation, self-reported previous diagnosis of IBS, or a hospital primary or secondary ICD-10 diagnosis. Patients with IBD, celiac disease, or those who underwent prior bowel resection were excluded to prevent signal contamination. Genome control was applied to age, sex, and the first 20 major components of the available genetic data. The GWAS statistics of IBS used in the current study contains cohorts of different IBS definitions. However, the incorporated datasets have been proved to show robustness irrespective of the particular case definition. The genetic correlation, or coheritability, between these various case definitions within the UK Biobank ranges from 70 to 100%, and the genetic correlation between UK Biobank and Bellygenes was also exceptionally high (99.8%) (23). Therefore, bias from varying diagnostic criteria can be maximally avoided.

Based on the meta-analysis by Liu et al. (24, 25),^2^ only 34,652 subjects of European descent were included in the GWAS statistics for IBD (cases/controls of IBD: 12,882/21,770; CD: 5,956/14,927; UC: 6,968/20,464) (Figure 1B). IBD GWAS samples used in Liu et al. were summarized in Supplementary Table S3. All included cases were diagnosed by accepted radiologic, endoscopic, and histopathologic evaluations in the International Inflammatory Bowel Disease Genetics Consortium (24, 26). The GWAS data were examined for associations with PLINK and modulated for 10, 7, or 15 of the first 20 principal components selected for CD, UC, or IBD, respectively.

To avoid weak IVs, genetic IVs were extracted from SNPs with *p*-values <5 × 10^−8^ (*p*-values <5 × 10^−6^ for IBS). Furthermore, TwoSampleMR::clump_data implementation (clump_r2 = 0.001, clump_kb = 100,000) was performed to remove variants with linkage disequilibrium.

### 2.2. Sources of SNP outcome data

SNP outcome data were obtained from two independent databases: large-scale GWAS meta-analysis data (23, 24), as described above, and FinnGen cohort (27)^3^ (Figure 1B). In the FinnGen cohort, the case definition was based on discharge or death, according to the ICD code. Additionally, IBD included CD, UC, and indeterminate colitis. GWAS data from the FinnGen project were analyzed and adjusted for batch effects based on age, sex, principal component, and genotype.

### 2.3. MR analysis

Prior to analysis, the exposure and outcome data were harmonized to ensure that each IV was aligned with the same effect alleles (28). The relationships between genetic tools and putative risk factors were quantified using F-statistics (*F* > 10) (29, 30). The phenotype variance attributable to SNPs was derived from the Mangrove package in R (31). The method described by Brion et al. was used to check the statistical power (32), with the alpha level set to 0.05.

Inverse variance weighted (IVW) MR is a powerful method with valid IVs and balanced pleiotropy assumptions (33). The causality and reverse causality estimates between IBS and IBD were based on IVW as the primary analysis method. In addition, several other MR methods [MR-Egger regression (34) and median-based (35) and mode-based (36) methods] were used to evaluate the reliability of our results. An effect was considered robust if the directions obtained using these methods were consistent.

Three assumptions underlie the MR analysis (Figure 1A). To determine whether the SNPs showed heterogeneity, Cochran’s Q and I^2^ statistics were performed. If heterogeneity was present, the multiplicative random-effects IVW model was adopted. Moreover, the MR Steiger test was used to detect reverse causality. MR Steiger hypothesizes that for a valid IV, there should be more variation in exposure than in outcome. Various methods were performed to minimize errors caused by level diversity, including correlation and unrelated-level pleiotropy. MR Pleiotropy RESidual Sum and Outlier (MR-PRESSO) and MR-Egger intercept tests (37) were both applied to evaluate relative pleiotropy. The MR-PRESSO outlier test (*p* < 0.05) detects significant outliers and eliminates horizontal pleiotropy effects if the test indicates horizontal pleiotropy across all instruments. Sensitivity was assessed using leave-one-out analysis to validate the robustness of the MR results.

For each binary exposure, an odds ratio (OR) with a 95% confidence interval (95% CI) was used to present the risk of each outcome per log-OR increase. An initial MR analysis was calculated separately per outcome study, followed by a fixed-effect meta-analysis combining individual estimates. Unless specified otherwise, *p* < 0.025 (0.05/2 databases) was used as a significance level after Bonferroni correction to account for multiple testing, with a 0.025 ≤ *p* < 0.05 being considered suggestive of significance.

R package TwoSampleMR (Version 0.5.5) was used for IVW MR, weighted median, simple median, MR-Egger (Egger regression), and leave-one-out analyses. R package Mangrove (Version 1.21) was used to derive phenotype variance attributable to SNPs. MR-PRESSO (Version 1.0) outlier test was conducted using R package MR-PRESSO. And the “meta” (Version 6.0–0) package was used for meta-analysis.

## 3. Results

### 3.1. No causal associations of IBS and IBD

In the context of IBS, 41 independent SNPs were identified as genetic instrumental variable, with a median (minimum, maximum) F statistic of 23.3 (20.9, 37.0) (Supplementary Table S4). All SNPs had F statistics greater than 10, indicating no underlying weak instrumental bias. Details of the 41 SNPs are listed in Supplementary Table S5. An expected OR of ≥ 1.208 for IBD, ≥ 1.285 for CD, and ≥ 1.254 for UC in IBS was detected with 80% power (Figure 1B).

**Figure 2:**
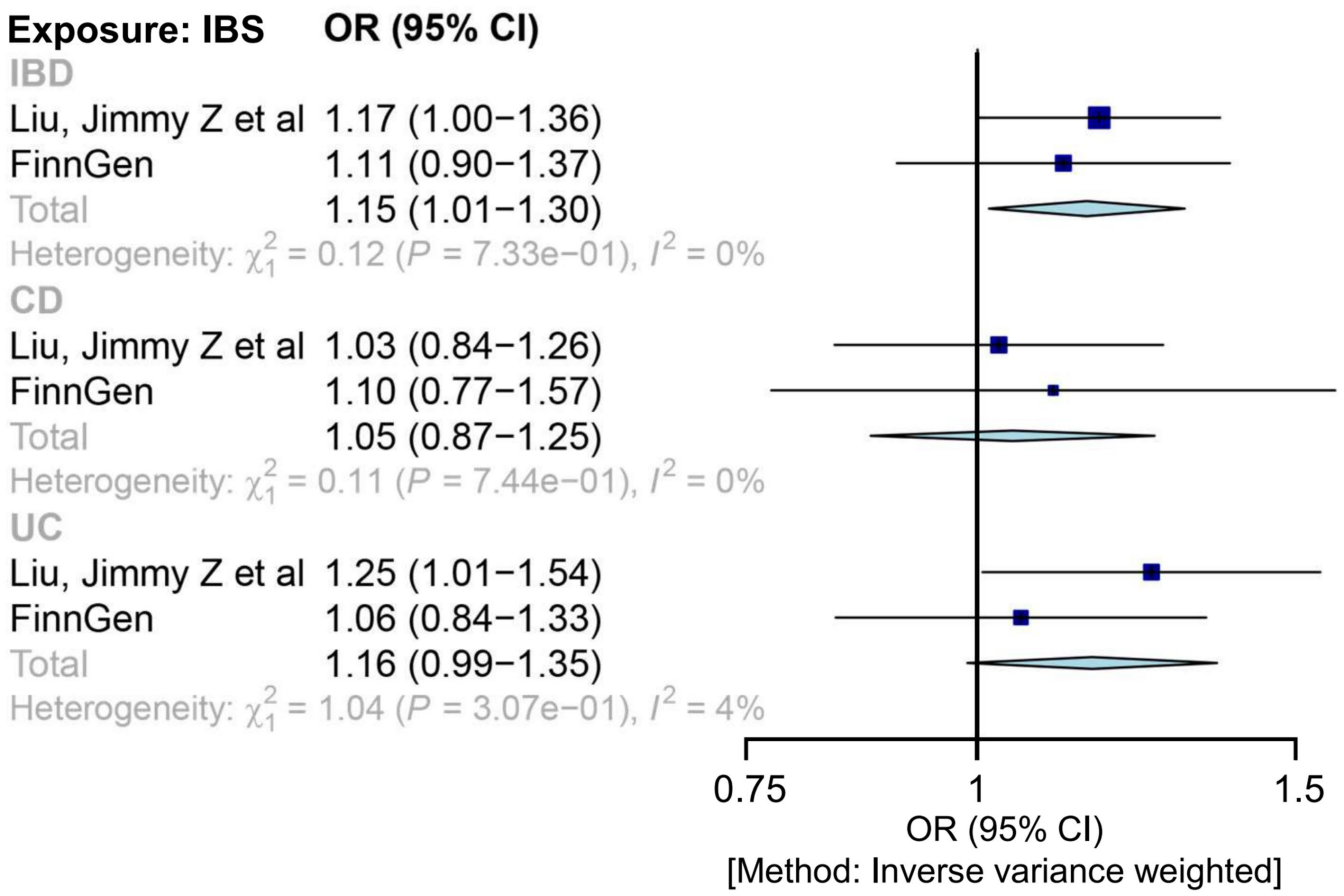
Mendelian randomization estimates for the relationship between genetically instrumented IBS and IBD, after meta-analyzing the results of inverse-variance weighted methods. CD, Crohn’s disease; CI, confidence interval; IBD, inflammatory bowel disease; IBS, irritable bowel disease; OR, odds ratio; UC, ulcerative colitis.

Applying the IVW MR analysis, there were no causal associations between genetically liable IBS to IBD in any outcome data, except in the IBD GWAS (Liu et al.), which showed mild correlations of IBS with IBD (1.17 (1.00, 1.36); *p* = 0.049) and UC (1.25 (1.01, 1.54); *p* = 0.044) (Supplementary Table S6). The combined ORs of IBS for IBD, CD, and UC were 1.15 (1.01, 1.30), 1.05 (0.87, 1.25), and 1.16 (0.99, 1.35), respectively (Figure 2). In each outcome data, our results using other MR methods also showed similar correlative trends (Supplementary Table S6 and Supplementary Figure S1).

Significant heterogeneity was observed for IBD only in the FinnGen cohort (Supplementary Table S7). A polymorphic effect was detected for IBD and UC in the FinnGen cohort (Supplementary Table S7). Therefore, we conducted an MR-PRESSO analysis after the outlier removal (Supplementary Table S6). The IBD results from the FinnGen cohort remained similar after the outlier correction. Furthermore, this association could not be explained by a single extreme SNP, as shown by leave-one-out analysis (Supplementary Figure S2). Steiger filtering indicated that the IVs used for IBS did not provide a greater explanation of variance for any IBD GWAS.

### 3.2. IBD is causally related to IBS

For IBD, 52 independent SNPs were identified as genetic IVs, compared to 47 for CD and 30 for UC. All SNPs had F statistics greater than 30, above the standard critical value (>10), indicating adequate instrument strength (Supplementary Tables S4, S8) (38). In the primary MR analysis, except for the impact of UC risk on IBS in the FinnGen cohort, the results show an 80% statistical power in detecting the impact of OR exposure liability on IBD from 1.01 to 1.06 (Figure 1B).

**Figure 3:**
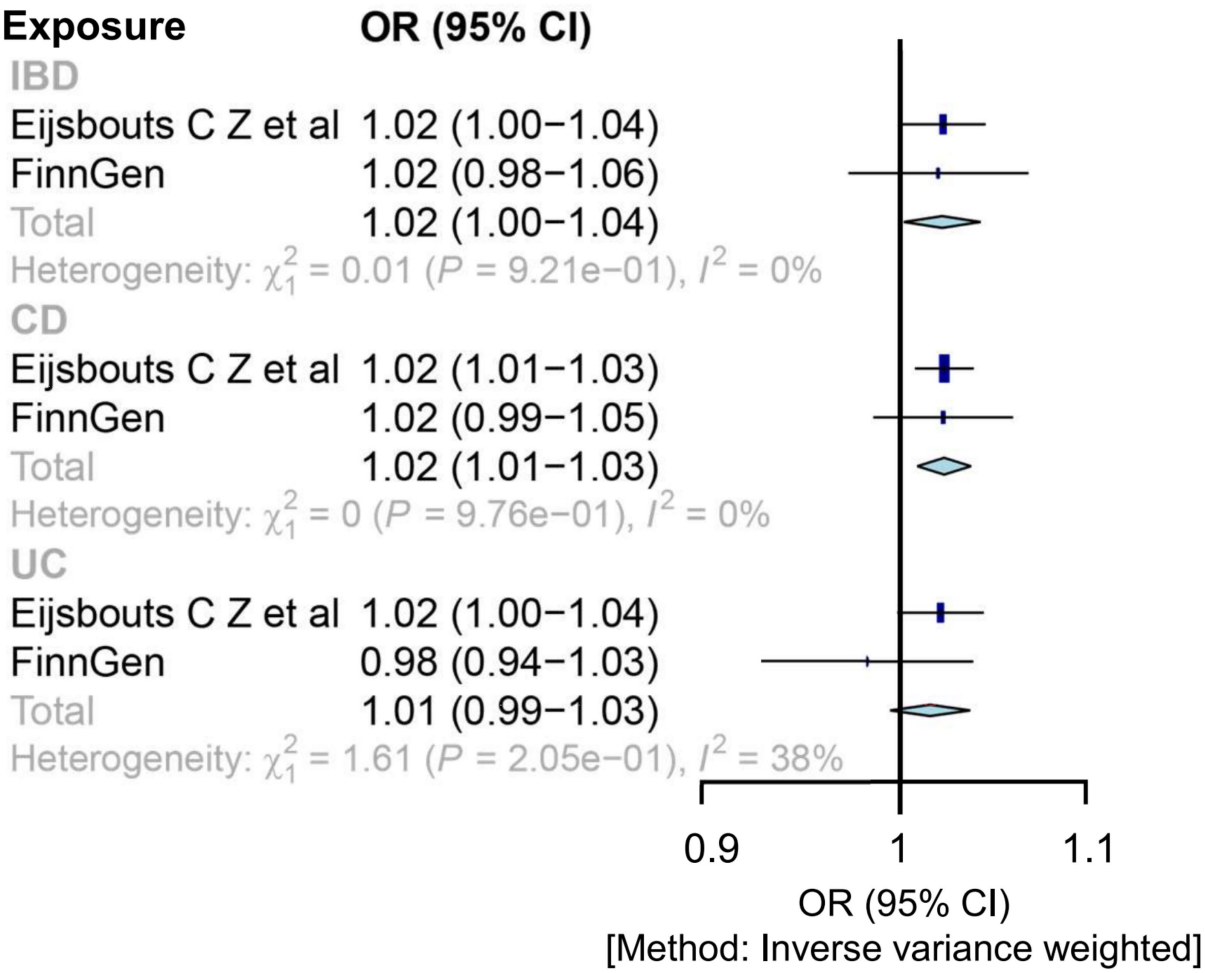
Mendelian randomization estimates for the relationship between genetically instrumented IBD and IBS after meta-analyzing the results of inverse-variance weighted methods. CD, Crohn’s disease; CI, confidence interval; IBD, inflammatory bowel disease; IBS, irritable bowel disease; OR, odds ratio; UC, ulcerative colitis.

In the meta-analysis of IVW estimates, the combined ORs of IBD and CD were 1.02 (1.00, 1.04) and 1.02 (1.01, 1.03), respectively (Figure 3 and Table 1). Additionally, other MR methods concurred with the IVW results in terms of the directions of association (Table 1 and Supplementary Figure S3).

**Table 1:** Effect estimates of the associations of genetic instrumental variables for inflammatory bowel disease and the risk of irritable bowel syndromes.

	No. of SNPs	IVW		Weighted Median		Multiplicative random effects IVW		MR-PRESSO		
								(outlier-corrected)		
		OR	*p-*value	OR	*p-*value	OR	*p-*value	Outlier	OR	p for
		(95% CI)		(95% CI)		(95% CI)			(95% CI)	global test
IBD										
Eijsbouts et al.	52	1.02 (1, 1.04)	0.057	1.02 (1, 1.05)	0.025	1.02 (1, 1.04)	0.057	1	1.02 (1.01, 1.04)	0.0008
FinnGen	42	1.02 (0.98, 1.06)	0.434	1.03 (0.98, 1.1)	0.251	1.02 (0.98,1.06)	0.434	0	NA	0.250
Meta-analysis		1.02 (1.00, 1.04)	**–**	1.03 (1.01, 1.05)	–	1.02 (1.00, 1.04)	**–**	**–**	**–**	**–**
CD										
Eijsbouts et al.	46	1.02 (1.01, 1.03)	0.004	1.02 (1, 1.03)	0.067	1.02 (1.01, 1.03)	0.004	0	NA	0.051
FinnGen	44	1.02 (0.99, 1.05)	0.247	1.03 (0.98, 1.07)	0.287	1.02 (0.99, 1.05)	0.247	0	NA	0.302
Meta-analysis		1.02 (1.01, 1.03)	–	1.02 (1.00, 1.03)	–	1.02 (1.01, 1.03)	–	–	–	–
UC										
Eijsbouts et al.	30	1.02 (1, 1.04)	0.079	1.02 (0.99, 1.04)	0.131	1.02 (1, 1.04)	0.079	1	1.03 (1.02, 1.05)	0.001
FinnGen	26	0.98 (0.94, 1.03)	0.508	0.99 (0.93, 1.05)	0.785	0.98 (0.94, 1.03)	0.508	0	NA	0.105
Meta-analysis		1.01 (0.99, 1.03)	–	1.01 (0.99, 1.04)	–	1.01 (0.99, 1.03)	–	–	–	–

Results of meta-analyses were obtained using the fixed-effect model. CD, Crohn’s disease; CI, confidence interval; IBD, inflammatory bowel disease; IVW, inverse variance weighted; MR-PRESSO, MR Pleiotropy RESidual Sum and Outlier; NA, not available; OR, odds ratio; UC, ulcerative colitis.

Cochran’s Q test and I^2^ statistics identified significant heterogeneity for the IBS GWAS (Eijsbouts et al.). The MR-PRESSO test detected horizontal pleiotropic outliers from both the general IBD to the IBS GWAS (Eijsbouts et al.) and the UC to IBS GWAS (Eijsbouts et al.), despite not being on the MR-Egger intercept test (Supplementary Table S9). After correcting for outliers using MR-PRESSO, there was a significant correlation [1.02 (1.01, 1.04); *p* = 0.00083; 1.03 (1.02, 1.05); *p* = 0.001] (Supplementary Table S9). Supplementary Figure S4 shows the leave-one-out analysis results of these associations. Steiger filtering showed that the IVs used for IBD did not provide a greater explanation of variance for the IBS GWASs.

## 4. Discussion

In this study, MR analysis was used to assess the bidirectional associations between IBS and IBD. IBS risk increased with genetic liability for IBD (such as CD and UC), while genetic liability for IBS was unrelated to IBD risk. Despite the application of random-effects IVW and the results of the sensitivity analysis, which suggest that our results were generally robust, the presence of heterogeneity in the IVs suggests that biological interpretations should be approached with caution.

The results showed that genetic liability for IBS was unrelated to IBD risk, except for suggestive statistics in UC (*p* = 0.043). Our results do not agree with those of observational studies. The diagnosis rate of IBD in IBS patients varies greatly among previously reported studies, with the probability of patients with IBS developing IBD ranging from 0.4 to 19% (38–44). The previously observed positive correlations between IBS and IBD may be coincidental or confounded by unknown factors rather than being directly caused by IBS. For example, Asghar et al. (45) found that the rate of IBD diagnosis via colonoscopy was statistically higher in patients with Rome IV FBD symptoms than in patients without (6.2% vs. 2.2%, *p* = 0.012); however, the family history of IBD differed significantly between the two groups (*p* = 0.02) (45). The prevalence of IBD was relatively low (0.4–2.1%) in the two studies of IBS that excluded patients with a family history of IBD (39, 43), while the remaining studies with a high IBD prevalence did not mention whether a first-degree relative had IBD, and the confounding effects of the included population were not removed (39–41, 43). As mentioned above, IBD is causally related to IBS, and familial IBD increases the risk of IBS. Moreover, IBD has a multifactorial pathogenesis, with genetic, environmental, and immunological factors all playing a role in its development and progression. Additionally, its high incidence may be due to a family history of IBD. We trust our results in this regard, which suggest that colonoscopy, with its additional expense and invasive testing, may not be necessary to diagnose and monitor patients with IBS, at least not for its relationship with IBD risk. In a meta-analysis, normative levels of C-reactive protein or fecal calcitonin were better negative predictors of IBD (46). It’s worth noting that in our results, the combined ORs with consistent confidence intervals only for IBS on IBD, but not for IBS on CD and IBS on UC. The possible explanation is that the genetic instrumental variables used may have stronger effects on some aspects of IBD shared between CD and UC, but weaker effects on the specific characteristics that distinguish CD and UC from each other. Such effects are amplified when CD and UC are combined as a whole, which may result in more consistent confidence intervals than when individual IBD, UC or CD, are analyzed separately. In addition, sample size is another factor that may affect confidence intervals. In general, IBD has a larger sample size than its subsets (UC or CD), and thus may have smaller and more consistent confidence intervals than individual IBD.

Our results confirmed a causal relationship of IBD on IBS, avoiding the inherent confounding and reverse causality of observational studies. Despite the strong correlation between the two disorders, the majority of previous studies utilized cross-sectional or case–control designs; therefore, causality has not been established. IBS-TSs, based on the Manning or Rome II, III, or IV criteria, were positively associated with IBD in a meta-analysis of 27 prospective observational studies (12). In the aforementioned study, there was also a greater risk of IBS-TSs in CD patients. A new prospective study bolstered these results by assessing the natural history and effect of IBS-TSs during IBD follow-up (47). In that study, IBS-TSs affected two-thirds of patients with IBD, demonstrating the effects of IBS on mental health and quality of life, although they did not significantly impact future disease outbreaks or adverse disease activity outcomes (i.e., glucocorticoid use, medication escalation, incidence of hospitalization, or surgery). The first-degree relatives of patients with IBD tended to suffer from IBS (48), suggesting a genetic link and the likelihood that these patients would be susceptible to concomitant IBS and IBD. The results of our MR study strengthen the evidence for causality and help clarify the direction of the relationship. Our results suggest that IBS-TSs occurring after IBD cannot be considered an active phase of IBD because IBD will cause IBS. This has significance for the diagnosis and treatment of patients with IBS occurring after IBD. Clinicians should be aware that initiating immunotherapy for IBS-TSs in patients with IBD carries risks of infection, malignant complications, and increased healthcare costs while offering little clinical benefit (49). Thus, our study provides quantitative theoretical evidence for concomitant IBD and IBS.

IBS development in IBD may result from disorders of the gut-brain axis, disturbed motility, and altered gut microbiota (6, 11). Thus, neuromodulators and gut-brain behavioral therapies are recommended in IBS guidelines (50), but it is worth noting that trials involving these treatments have shown disappointing results in patients with IBD (50–52). Few studies in the literature have focused on the specific role of psychotherapy in enhancing therapeutic adherence among patients with IBD (53). In one trial comparing IBS-TSs in patients with and without IBD, hypnotherapy did not yield better responses than standard drug therapy (51). A diet low in fermentable oligosaccharides, disaccharides, monosaccharides, and polyols (FODMAP) has proven to be effective for IBS (54). Additionally, a randomized controlled study confirmed that a 4-week low-FODMAP diet was safe, and improved intestinal symptoms in IBD patients with IBS-TSs (55). However, it is important to compare other treatments tailored to IBS in patients with IBD-induced IBS.

Some limitations exist in this study. First, the data mainly included European patients and are not necessarily applicable to other ethnic groups. However, this limitation minimizes potential population stratification bias. Second, rather than considering the course of IBD, the dichotomous diagnosis of IBD was examined. In IBD, remission and relapse occur alternately, and episodes are largely unpredictable. However, uncovering the genetic makeup associated with IBD activity remains challenging. Owing to the absence of an IBD course GWAS, the relevant cause-and-effect relationship cannot be confirmed by MR Methods. Third, subgroup analyses were not performed based on individual-level demographic and clinical parameter information due to the lack of individual-level data. However, the lack of individual-level data does not affect the results of the current study as the methods used were based on summary-level data. Finally, this study did not perform IBS typing, which is used as a binary variable in the GWAS, and did not consider varying symptoms. Therefore, the association between IBD and IBS may vary depending on the IBS classification.

## Data availability statement

The datasets presented in this study can be found in online repositories. The names of the repository/repositories and accession number(s) can be found in the article/Supplementary material.

## Ethics statement

Ethical review and approval was not required for the study on human participants in accordance with the local legislation and institutional requirements. Written informed consent for participation was not required for this study in accordance with the national legislation and the institutional requirements.

## Author contributions

YC and JC: conceptualization, supervision, funding acquisition. ZL, HK, QL, and ZS: data curation. ZL: writing—original draft preparation. ZL, HK, QL, JC, and YC: writing—review and editing. All authors contributed to the article and approved the submitted version.

## Funding

This work was supported by the Special Scientific Research Fund for the National Natural Science Foundation (grant nos. 82070650, 82070543, and 8177031240).

## Conflict of interest

The authors declare that the research was conducted in the absence of any commercial or financial relationships that could be construed as a potential conflict of interest.

## Publisher’s note

All claims expressed in this article are solely those of the authors and do not necessarily represent those of their affiliated organizations, or those of the publisher, the editors and the reviewers. Any product that may be evaluated in this article, or claim that may be made by its manufacturer, is not guaranteed or endorsed by the publisher.

## Supplementary material

The Supplementary material for this article can be found online at: https://www.frontiersin.org/articles/10.3389/fmed.2023.1166683/full#supplementary-material

## Footnotes

^1^ https://www.ibdgenetics.org/downloads.html

^2^ https://gwas.mrcieu.ac.uk/

^3^ https://www.finngen.fi/en/access_results

## Abbreviations

CD: Crohn’s disease
GWAS: genome-wide association studies
IBD: inflammatory bowel disease
IBS: irritable bowel syndrome
IBS-TSs: IBS-type symptoms
IV: instrumental variables
MR: Mendelian randomization
MR-PRESSO: MR Pleiotropy RESidual Sum and Outlier
OR: odds ratio
SNPs: single-nucleotide polymorphisms
UC: ulcerative colitis
95% CI: 95% confidence intervals
