# Supplementary material for: Inflammatory bowel disease is causally related to irritable bowel syndrome: a bidirectional two-sample Mendelian randomization study
Source: Front Med (Lausanne). 2023 Apr 17;10:1166683. doi: 10.3389/fmed.2023.1166683 (PMC10150057; doi:10.3389/fmed.2023.1166683)
Supplement: Supplementary file 1 [file Data_Sheet_1.docx]

Supplementary Material

Causal Relationship Between Inflammatory Bowel Disease and Irritable Bowel Syndrome: A Bidirectional Two-Sample Mendelian Randomization Study

Haoran Ke, Zitong Li, Qianyun Lin, Zefeng Shen, Ye Chen^⁎^, Jinjun Chen*

*** Correspondence:** Ye Chen: yechen@smu.edu.cn; Jinjun Chen: [chjj@smu.edu.cn](mailto:chjj@smu.edu.cn)

## Supplementary Figures


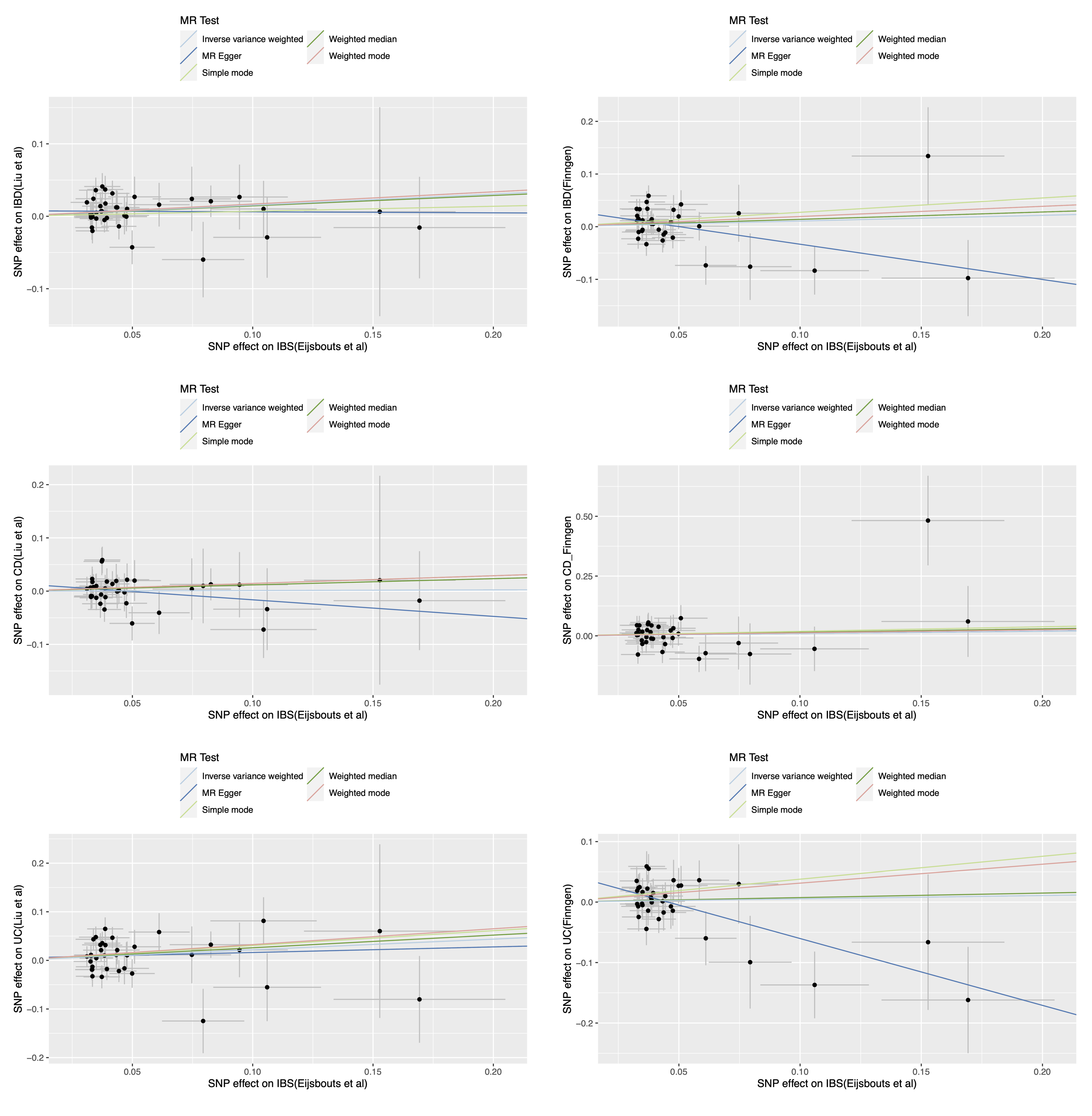


**Supplementary Figure 1.** Scatter plot of MR analyses from IBS to IBD in each database. The x-axes represent the genetic instrument-IBS associations and y-axes represent genetic instrument-IBD associations from different outcome databases. Black dots denote the genetic instruments included in the primary MR analyses. Red: inverse-variance weighted; blue: weighted-median estimator; green: MR Egger. IBS: irritable bowel symptoms; IBD: inflammatory bowel disease; CD: Crohn’s disease; UC: ulcerative colitis.


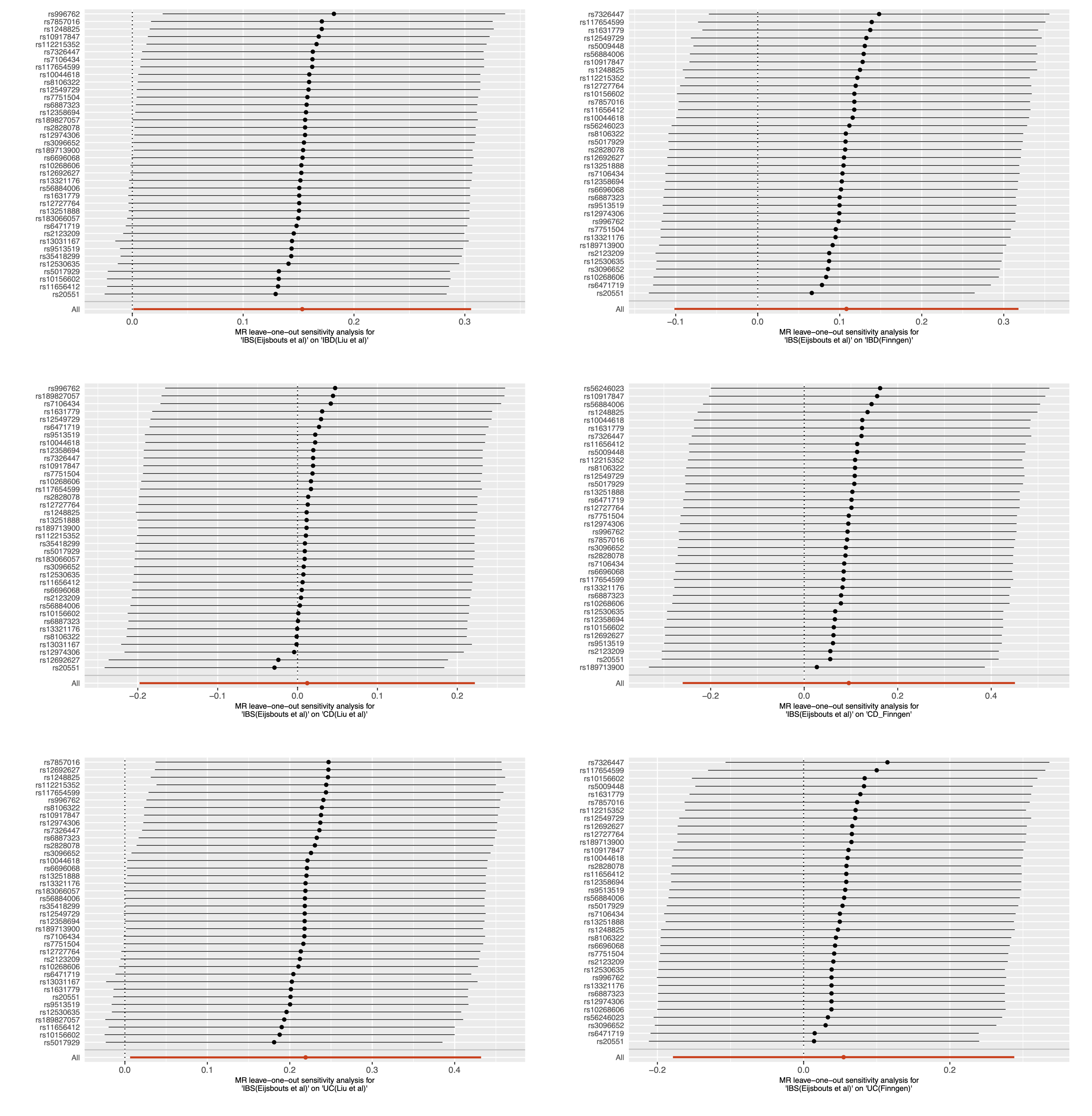


**Supplementary Figure 2.** Leave-one-out plot of MR analyses from irritable bowel symptoms to inflammatory bowel disease in each database

**
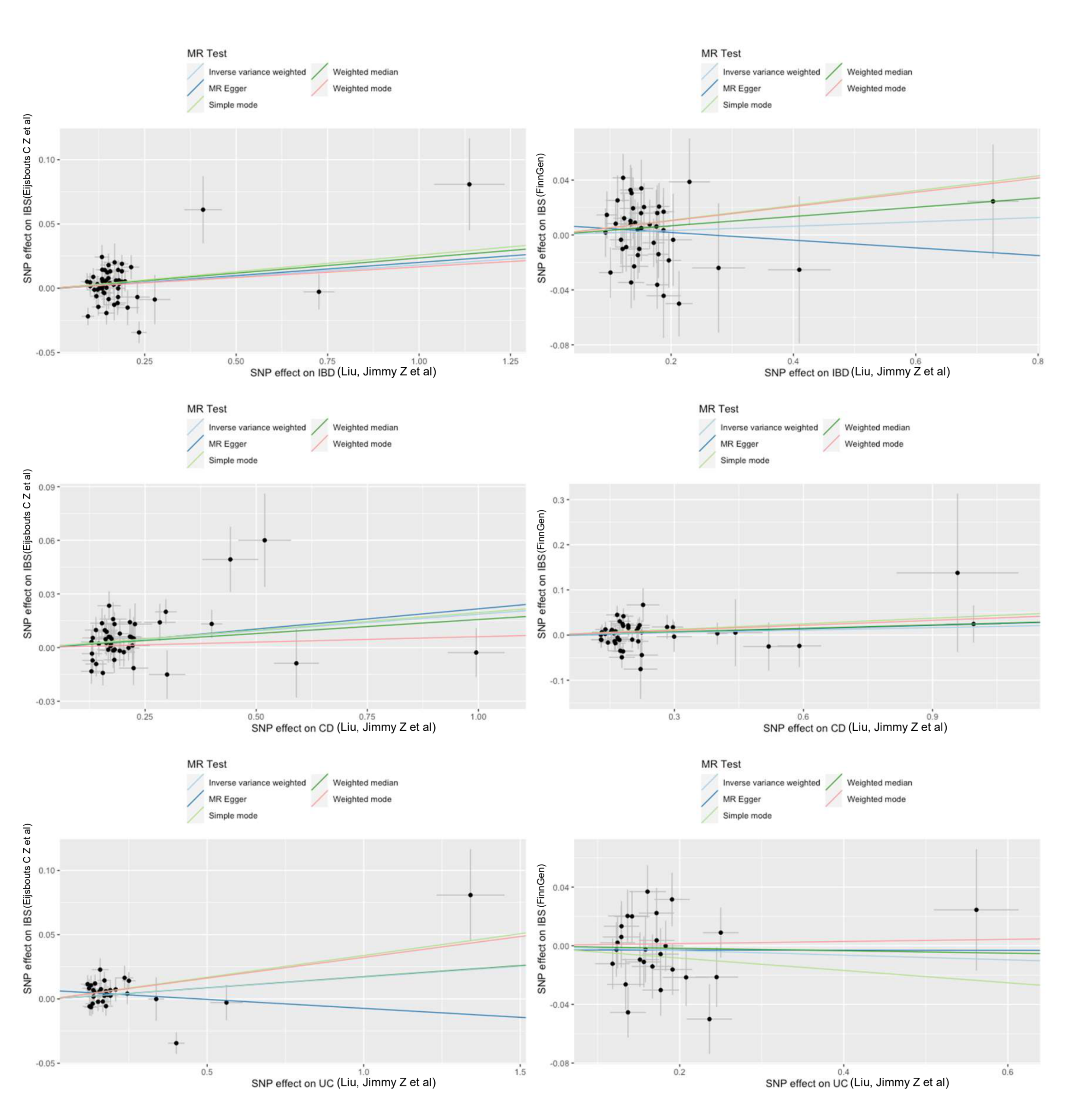
**

**Supplementary Figure 3.** Scatter plot of MR analyses from IBD to IBS in each database. The x-axes represent the genetic instrument-IBD associations and y-axes represent genetic instrument-IBS associations from different outcome databases. Black dots denote the genetic instruments included in the primary MR analyses. Red: inverse-variance weighted; blue: weighted-median estimator; green: MR Egger. IBS: irritable bowel symptoms; IBD: inflammatory bowel disease; CD: Crohn’s disease; UC: ulcerative colitis.


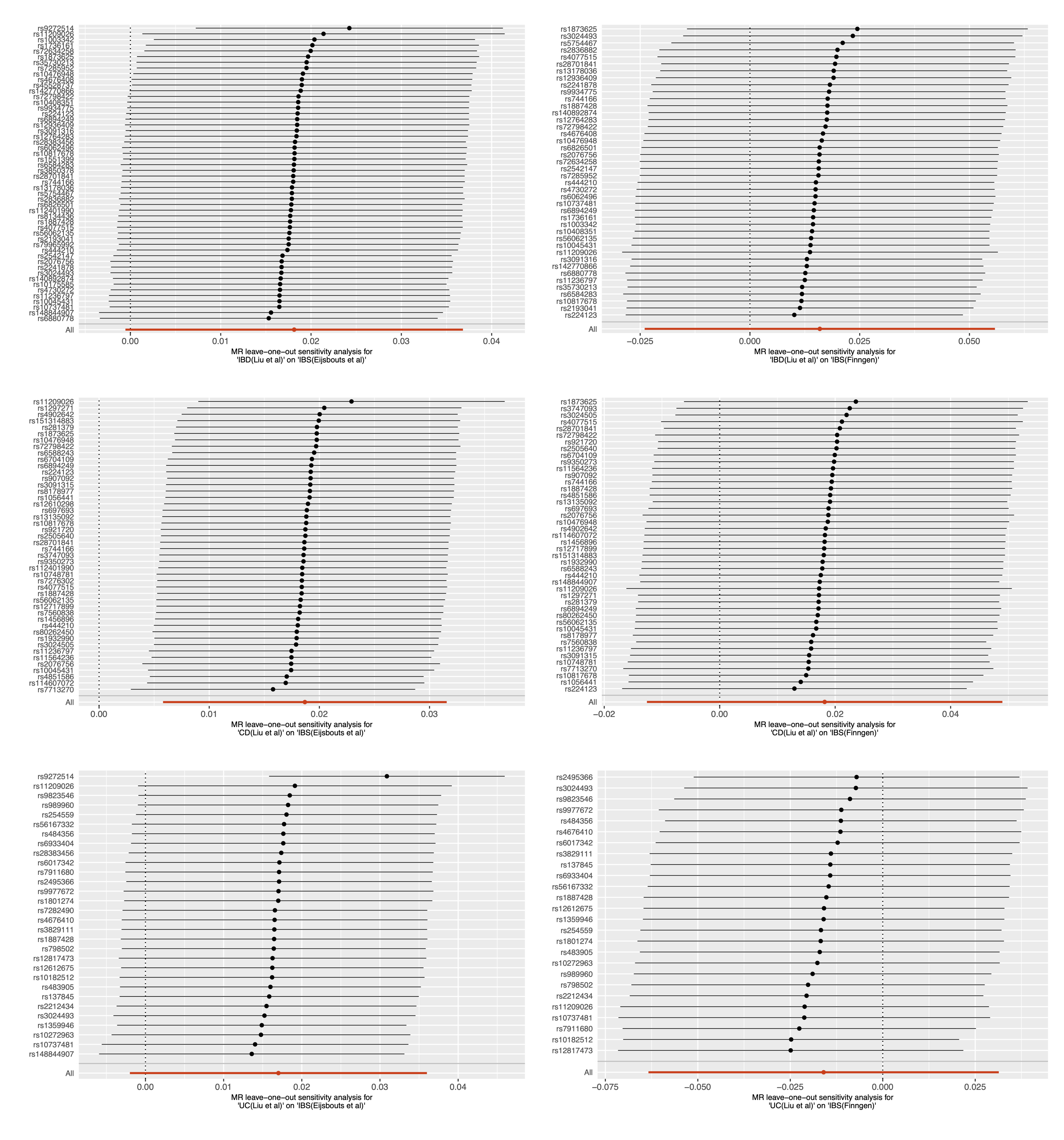


**Supplementary Figure 4.** Leave-one-out plot of MR analyses from inflammatory bowel disease to irritable bowel symptoms in each database.
